# KSHV ORF59 and PAN RNA Recruit Histone Demethylases to the Viral Chromatin during Lytic Reactivation

**DOI:** 10.3390/v12040420

**Published:** 2020-04-09

**Authors:** Kayla Hiura, Roxanne Strahan, Timsy Uppal, Brian Prince, Cyprian C. Rossetto, Subhash C. Verma

**Affiliations:** Department of Microbiology and Immunology, University of Nevada, Reno School of Medicine, 1664 N Virginia Street, Reno, NV 89557, USA; kaylahiura@gmail.com (K.H.); roxcrocks@gmail.com (R.S.); tuppal@med.unr.edu (T.U.); bcprince0@gmail.com (B.P.); crossetto@med.unr.edu (C.C.R.)

**Keywords:** KSHV, lytic reactivation, ORF59, PAN RNA, UTX, JMJD3

## Abstract

Kaposi’s sarcoma-associated herpesvirus (KSHV) causes multiple malignancies in immunocompromised individuals. KSHV primarily establishes a lifelong latency in infected humans during which only a subset of viral genes is expressed while most of the viral genome remains transcriptionally silent with condensed chromatin. However, during the lytic phase, the viral genome undergoes dramatic changes in chromatin landscape leading to a transcriptionally active state with the expression of most of the viral genes and production of progeny virions. Multiple cellular and viral factors influence the epigenetic gene regulation and transitioning of virus from latency to the lytic state. We have earlier shown that KSHV ORF59, viral processivity factor, binds to a protein arginine methyl transferase 5 (PRMT5) to alter the histone arginine methylation during reactivation. Additionally, ORF59 has been shown to interact with most abundantly expressed KSHV long noncoding polyadenylated nuclear RNA (PAN RNA), which associates with the viral epigenome during reactivation. Interestingly, PAN RNA interacts with UTX and JMJD3, cellular H3K27me3 demethylases, and removes the repressive marks on the chromatin. In this study, we report that the recruitment of histone demethylases to the viral chromatin is facilitated by the expression of ORF59 protein and PAN RNA. Using biochemical and localization assays including co-immunoprecipitation and immunofluorescence, we demonstate ORF59 localizes with UTX and JMJD3. Our results confirm that PAN RNA enhances the interaction of ORF59 with the chromatin modifying enzymes UTX and JMJD3.

## 1. Introduction

Kaposi’s sarcoma-associated herpesvirus (KSHV), an oncogenic human γ-herpesvirus, is the cause of Kaposi’s Sarcoma [1,2,3], primary effusion lymphoma [4], KSHV inflammatory cytokine syndrome [5], and some forms of multicentric Castleman’s disease [6,7]. KSHV establishes a life-long dormant (latent) infection in infected individuals, during which only a limited number of viral genes, required for maintaining the virus in the infected host and passing the viral genome following replication into the divided tumor cells, are expressed (reviewed in [8]). During latency, the viral genome exists in a chromatinized state, enriched with both the “activating (AcH3, H3K4me3)” and “repressive (H3K27me3)” histone modifications, which promote latent gene expression over the lytic gene expression [9,10]. The latently infected cells can undergo lytic reactivation to produce infectious virions in response to a variety of chemical agents or to replication and transcriptional activator (RTA), an IE and lytic switch protein, that triggers the lytic cascade [11,12,13,14,15]. During reactivation, nearly all the viral genes (~90) are expressed in a synchronized manner, with the expression of immediate early (IE), early (E), and late (L) genes, leading to the amplification of viral DNA followed by packaging and release of virion particles [6,16]. Interestingly, this entire process starts with a change in the epigenetic architecture of viral promoters through the involvement of multiple viral and cellular proteins [6,10,17,18].

KSHV is epigenetically controlled, as the promoter of latent-to-lytic switch protein RTA exists as a bivalent chromatin enriched in both activating (AcH3, H3K4me3) and repressive (H3K27me3) epigenetic marks [9,17]. Components of the polycomb repressive complex 2 (PRC2)-EZH2, SUZ12, EED, and RbaAp48/46 are shown to associate with the repressive marks, specially the H3K27me3 on the KSHV genome [9]. The polycomb repressive complex 1 (PRC1), which represses transcription by monoubiquitinating lysine 119 of H2A (H2AK119ub), also associates with the chromatin of RTA promoter [19]. EZH2 dissociates from the RTA promoter along with a decrease in H3K27me3 and subsequent increase in acetylation of histone H3 (AcH3) and methylation of H3K4 (H3K4me3) during lytic reactivation [19]. Two key important viral factors that have been implicated in epigenetic gene regulation of KSHV during the lytic reactivation are KSHV ORF59 protein, a viral processivity factor that helps in DNA replication by complexing with viral DNA polymerase [20,21], and a lytic viral polyadenylated long noncoding RNA (PAN RNA) (Reviewed in [22]), produced at high levels in the nucleus of infected cells [23].

We recently reported that ORF59 protein can alter the chromatin landscape by modifying the arginine methylation state of histone H4 [24]. A reduction in the symmetric methylation of H4R3 (H4R3me2s) results in the elimination of repressive chromatin marks and the enrichment of activating marks on the viral chromatin, leading to the activation of lytic cascade [24]. We also earlier demonstrated that PAN RNA interacts with host demethylases UTX and JMJD3 to remove the repressive H3K27me3 mark from the viral chromatin [25]. We found that PAN RNA alters immune response by manipulating the expression of genes that modulate immune response [26]. Additionally, ORF59 was shown to interact with PAN RNA in RNA affinity pull-down assay and also found to associate with the chromatin of the KSHV genome [24,27]. Here, we show that PAN RNA acts as a scaffold in binding of the histone-modifying enzymes to ORF59. This interaction could be further exploited for controlling the lytic reactivation. In the studies presented here, we investigated if ORF59 and PAN RNA recruit histone modifying enzymes, UTX and JMJD3, at specific regions of the viral genome. Immunoprecipitation and immunostaining analysis of infected or transfected cells demonstrated that the recruitment of histone demethylases to the viral chromatin is facilitated by the expression of ORF59 protein and PAN RNA. We also showed that ORF59 colocalizes with UTX and JMJD3 proteins in the same nuclear compartments at many loci. Our results confirmed that PAN RNA plays an important role in the epigenetic regulation of the KSHV genome by facilitating ORF59-UTX/JMJD3 association during lytic replication.

## 2. Materials and Methods

### 2.1. Cell Culture

HEK293 cells (ATCC, Manassas, VA) were maintained in high-glucose Dulbecco’s modified Eagle’s medium (DMEM) supplemented with 8% bovine growth serum (HyClone, Logan, UT), 2 mM L-glutamine, 25 U/mL penicillin, and 25 μg/mL streptomycin. iSLK/BAC16 cells with HA-tagged ORF59 cells were grown in DMEM supplemented with 10% tet-free fetal bovine serum with 600 μg/mL hygromycin B, 400 μg/mL G418 and 1 μg/mL puromycin. The cells were induced with 0.3 M NaB (sodium butyrate, Sigma-Aldrich, St. Louis, MO, USA) and 1 ug/mL doxycycline (Sigma-Aldrich, St. Louis, MO). All cell lines were grown in a humidified environment at 37 °C supplemented with 5% CO_2_.

### 2.2. Antibodies, Chemicals and Plasmids

The following plasmids, antibodies and oligos were used: rabbit anti-HA (6908, Sigma-Aldrich, St. Louis, MO, USA), mouse anti-HA(12CA5) (sc-57592, Santa Cruz Biotechnology, Santa Cruz, CA, USA), mouse anti-GFP (G1546, Sigma-Aldrich, St. Louis), mouse anti-GAPDH (G8140, US Biological, Salem, MA, USA), mouse anti-ORF59 (gift from Dr. Bala Chandran), rabbit anti-control IgG (sc-2027, Santa Cruz Biotechnology), mouse anti-control IgG (sc-2025, Santa Cruz Biotechnology), rabbit anti-JMJD3 (3457S, Cell Signaling Technology, Danvers, MA, USA), rabbit anti-UTX (33510S, Cell Signaling Technology), control LacZ oligo (Protein and Nucleic Acid Facility, Stanford University), and PAN oligos (Protein and Nucleic Acid Facility, Stanford University). Plasmids pCS2-UTX-Flag and pCS2-JMJD3-Flag were purchased from Addgene.

### 2.3. DNA Transfection

HEK293 cells were transfected with 20 ug of respective DNA using linear polyethylenimine (pEI, Polysciences, Inc., Warrington, PA, USA) at a 1:3 pEI/DNA ratio. At 24 h post transfection, cells were harvested and lysed for western blot analysis, as mentioned below. 

### 2.4. Co-Immunoprecipitations and Western Blotting

After 24 h transfection, HEK293 or induced iSLK/Bac16-ORF59HA cells were washed with ice-cold PBS and lysed in 500 uL of ice-cold 1% NP-40 lysis buffer (50 mM Tris-HCl pH 7.5, 150 mM NaCl, 1% NP-40, 1 mM EDTA pH 8.0), supplemented with protease inhibitors (1 mM phenyl-methylsulfonyl fluoride, 1 μg/mL aprotinin, 1 μg/mL pepstatin, 1 μg/mL sodium fluoride, and 1 μg/mL leupeptin), followed by incubation on ice for 30 min. The lysate was centrifuged at 13,000× *g* for 10 min to remove cell debris and precleared by addition of 30 μL of Protein A-Protein-G-conjugated Sepharose beads to the lysate and rotation for 30 min at 4 °C. For RNAse treated samples, the lysate was incubated on ice with 1 uL of RNAse A for an additional 30 min before preclearance. About 5% of the lysate was saved as an input control and 1 μg of antibody was added to the remaining lysate and rotated overnight at 4 °C to capture the protein. The protein complexes were captured by adding 30~μL of Protein A/G-conjugated Sepharose beads and rotating the lysate for 2 h at 4 °C. The lysate was centrifuged at 2000× *g* for 2 min to pellet the beads and washed three times in 1% NP-40 Lysis Buffer. Input and immunoprecipitated lysates were boiled at 95 °C for 5 min in Laemmli buffer. The proteins were resolved on SDS-PAGE and transferred onto a 0.45 uM nitrocellulose membrane (Bio-Rad Laboratories, Hercules, CA, USA) using standard procedures. The membranes were incubated with primary antibodies followed by secondary infrared-dyed tagged antibodies and imaged on an Odyssey imager (LICOR Inc., Lincoln, NE, USA).

### 2.5. Immunofluorescence Assay

Cells were fixed in 3%–4% paraformaldehyde, permeabilized with 0.2% Triton X-100 for 10 min, and blocked with fish skin gelatin (FSG) blocking buffer (0.4% FSG, 0.05% Triton X-100) for 40 min at room temperature. The cells were then incubated with specific primary antibodies (0.5 ug) in 0.2% FSG/0.05% Triton X-100) overnight at 4 °C, washed with PBS, and incubated with Alexa Fluor conjugated secondary antibodies (0.2% FSG/0.05% Triton X-100) for 1 h at 37 °C. Nuclear staining was performed using TO-PRO3/PBS in PBS for 1 min. Cells were visualized and imaged using a confocal laser-scanning microscope (Carl Zeiss, Inc., San Diego, CA, USA) and processed with ZEN imaging software (Carl Zeiss, Inc.).

### 2.6. Quantitative Real-Time PCR (qRT-PCR)

Total mRNAs were extracted from the transfected cells using an Illustra RNAspin minikit (GE Healthcare). cDNAs were made using a high-capacity RNA-to-cDNA kit (Applied Biosystems Inc., CA, USA) as per the manufacturer’s protocol. The PCR reactions were made with 5 uL of sterile-water-diluted cDNA, 5 uL of forward and reverse primers (0.5 uM), and 10 uL of SYBR Green Universal master mix (Bio-Rad Laboratories) to a total of 20 uL. Primers for the GAPDH housekeeping genes were used for normalizing the threshold cycle (CT) values and relative gene copy numbers were calculated by the ΔΔCT method. All the reactions were run in triplicate.

### 2.7. Chromatin Isolation by RNA Purification (ChIRP) Assay

The ChIRP assay was performed using the method described earlier, with slight modifications [28]. Briefly, 20 million transfected HEK293 cells were harvested after 24 h and cross-linked with 0.5% formaldehyde for 15 min at room temperature, followed by addition of 125 mM glycine to halt the cross-linking. The cells were washed thrice with ice-cold PBS with protease inhibitors (1 μg/mL leupeptin, 1 μg/mL aprotinin, 1 μg/mL sodium fluoride, 1 μg/mL pepstatin, and 1 mM phenylmethylsulfonyl fluoride) and lysed in 1% NP-40 lysis buffer with protease inhibitors. The cells were then sonicated at 30 amps for 1 min to shear DNA fragments to an average length of 500–700 bp and centrifuged at 5000 rpm for 8 min to remove debris, and input samples were collected. Hybridization buffer (750 mM NaCl, 50 mM Tris.HCl pH 7.0, 1 mM EDTA, 1% SDS, 15% Formamide, water, 1 mM AEBSF, PIC, RNase inhibitor) was added to the remaining lysate in a 1:3 ratio of the lysis buffer. An quantity of 100 pmol of oligos was added and rotated for 4 h at 37 °C to capture the RNA interacting with the protein. Streptavidin beads were added to collect the RNA/protein complexes for 20 min, and the solution was centrifuged at 2000× *g* for 5 min to collect the beads, which were washed with washing buffer (2X SSC, 0.5% SDS, water, 1 mM AEBSF) 5 times. The beads were resuspended in 1% NP-40 lysis buffer and 3X PAGE Buffer. The input and treated samples were boiled for 10 min at 95 °C and resolved on SDS-PAGE.

## 3. Results 

### 3.1. ORF59 Interacts with Endogenous Demethylases UTX and JMJD3 in KSHV-Harboring Cells 

KSHV encodes several important lytic factors that are involved in the transitioning of latent virus to the lytic phase. These factors initiate KSHV’s lytic phase by altering the chromatin landscape from a “condensed/transcriptionally inactive chromatin” to an “open/transcriptionally active chromatin” that favors DNA transcription and replication [18]. Two cellular histone demethylases, primarily, UTX and JMJD3, are shown to remove the repressive H3K27me3 histone marks from the viral chromatin leading to an increase in the activating H3K4me3 histone marks to promote lytic reactivation [25]. These chromatin remodeling proteins are shown to interact with highly abundant, lytic long noncoding RNA (lncRNA) referred to as polyadenylated nuclear RNA (PAN RNA) expressed during lytic reactivation [23]. Previous studies have demonstrated that UTX and JMJD3 interact with PAN RNA [25], which has been shown to interact with ORF59 [27]. Here, we investigated if PAN RNA expression enhances the the binding of KSHV lytic protein ORF59 with these H3K27me3-specific histone demethylases.

**Figure 1:**
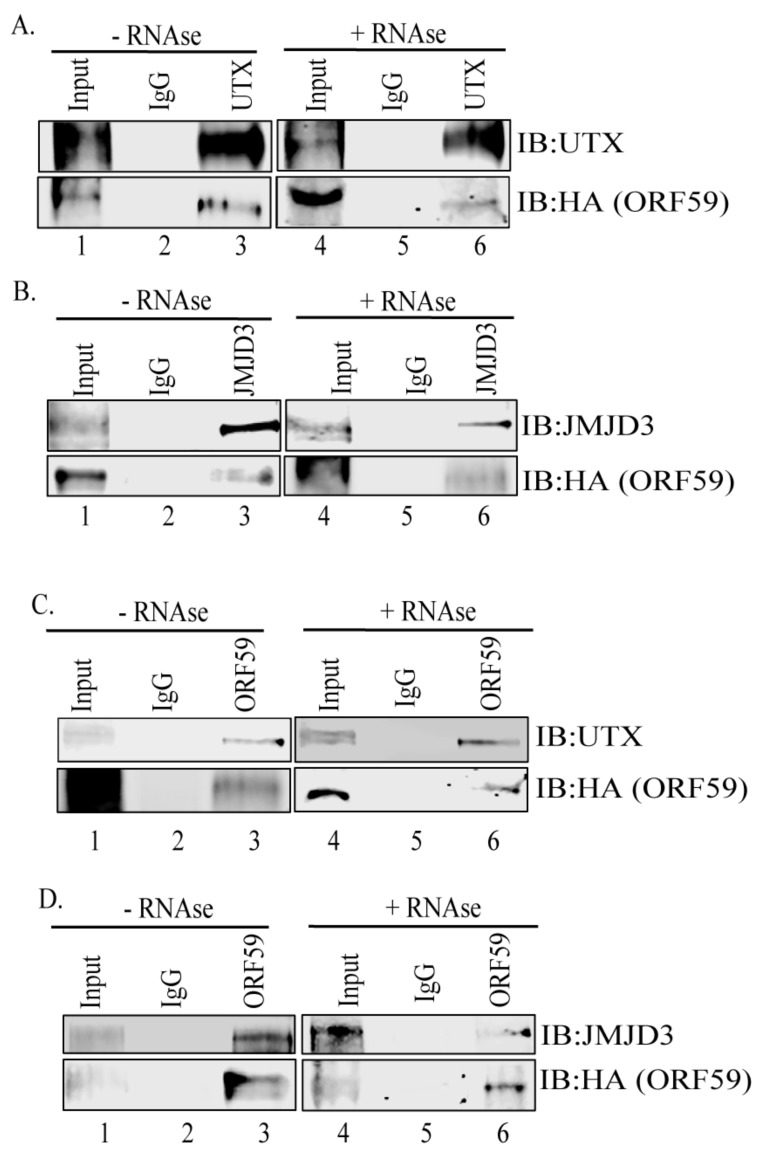
ORF59 binds to H3K27me3-specific demethylases UTX and JMJD3. (**A**) ORF59 was co-precipitated from iSLK/Bac16-ORF59HA cells using anti-UTX antibody (lane 3). Lysates from iSLK/Bac16-ORF59HA cells treated with RNase and precipitated using anti-UTX antibody showed binding between ORF59 and UTX. (**B**) ORF59 co-precipitated from iSLK/Bac16-ORF59HA cells using anti-JMJD3 antibody (lane 3). Lysates from iSLK/Bac16-ORF59HA cells treated with RNase and precipitated using anti-JMJD3 antibodies showed binding between ORF59 and UTX proteins. (**C**) Endogenous UTX protein was co-precipitated from KSHV-positive iSLK/Bac16-ORF59HA cells using anti-ORF59 antibody (lane 3). Lysates from KSHV-positive iSLK/Bac16-ORF59HA cells treated with RNase and precipitated using anti-ORF59 antibody showed binding between ORF59 and UTX. (**D**) JMJD3 protein was co-precipitated from iSLK/Bac16-ORF59HA cells using anti-ORF59 antibody (lane 3), and the lysates treated with RNase also showed binding between ORF59 and JMJD3 proteins.

To investigate the interaction between ORF59 and UTX or JMJD3 in KSHV-harboring cells, a co-immunoprecipitation (Co-IP) assay was performed on the endogenous proteins from KSHV-infected, 24 h-induced iSLK/Bac16-ORF59HA cells. Immunoprecipitations (IPs) were performed by adding control IgG or UTX or JMJD3 specific antibodies to the cell lysate. IP with anti-UTX antibody and detection of ORF59 showed that UTX precipitated ORF59 from the KSHV-infected cells (Figure 1A, lane 3). The lack of coprecipitating ORF59 protein from iSLK/Bac16-ORF59HA cells treated with anti-IgG control antibody confirmed the specificity of the UTX’s association with ORF59 (Figure 1A, lane 2). In addition, treatment of the lysates with RNAse to eliminate binding due to any inter-linking RNA still showed their association (Figure 1A, lane 6) suggesting that these two proteins interact directly. 

Similarly, IP with anti-JMJD3 antibody, followed by detection with ORF59 antibody, showed JMJD3′s interaction with ORF59 (Figure 1B, lane 3). The specificity of this interaction was confirmed by the lack of coprecipitating ORF59 from control IgG antibody (Figure 1B, lane 2). Samples treated with RNAse also showed ORF59′s interaction with JMJD3 [Figure 1B, lane 6] although possibly at lower levels as compared to samples without RNAse treatment suggesting that these proteins can interact directly.

In order to further determine the specificity of this interaction, a reverse co-IP assay was performed using anti-ORF59 antibody followed by detection with anti-UTX or anti-JMJD3 antibodies from iSLK/Bac16-ORFF59HA cells (Figure 1C,D). IP and subsequent detection with anti-ORF59 antibody confirmed that both UTX and JMJD3 efficiently precipitate ORF59 from these KSHV-positive cells (Figure 1C,D, lane 3). These results indicate that ORF59 interacts with endogenous histone modifying enzymes, UTX and JMJD3 in KSHV-infected cells.

### 3.2. ORF59 Co-Localizes with UTX and JMJD3 in KSHV-Positive Cells During Lytic Reactivation

**Figure 2:**
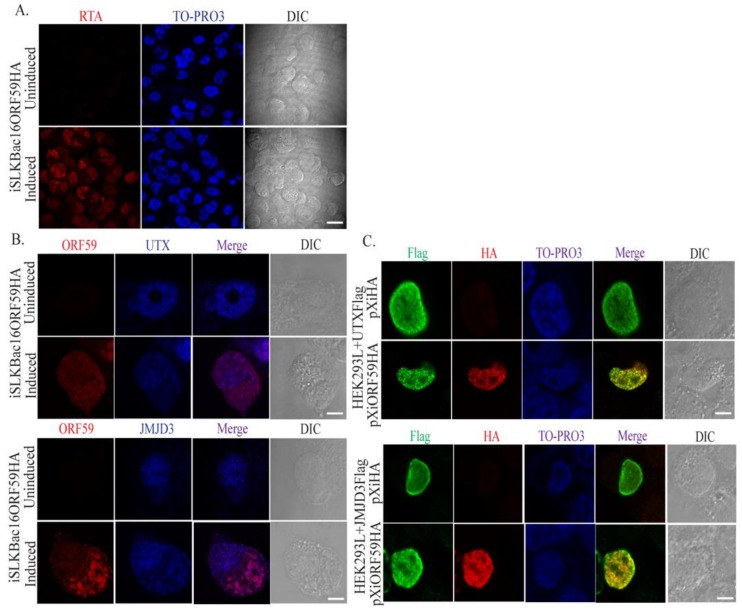
ORF59 co-localized with UTX and JMJD3 in the nuclear compartments. (**A**) Immunofluorescence analysis for RTA protein detection in 24 h-induced iSLK/Bac16-ORF59HA cells indicated almost all the cells underwent lytic reactivation; scale bar: 100 μm. The 24 h-induced iSLK/Bac16-ORF59HA cells (**B**) or transfected HEK293 cells (**C**) were stained with antibodies against ORF59, JMJD3, and UTX. ORF59 and JMJD3 or UTX localized at many foci in the nucleus of the induced cells (merge signal); scale bar: 20 μm.

In order to further validate the interactions between ORF59 and UTX or JMJD3, immunofluorescence assays (IFAs) using either 24 h-induced iSLK/BAC16-ORF59HA cells or transfected HEK293 cells (overexpression system) were performed to determine if these proteins localized in the same nuclear compartment in the infected cells during viral reactivation. IFA for RTA expression in iSLK/Bac16-ORF59HA cells plated on UV-treated coverslips, induced for 24 h and stained with anti-RTA (Figure 2A, red signal) indicated efficient lytic reactivation. Analysis of ORF59, UTX or JMJD3 expressions in induced iSLK/Bac16-ORF59HA cells using specific anti-ORF59, anti-UTX, and anti-JMJD3 antibodies indicated ORF59, a nuclear protein, showed a typical nuclear localization pattern (Figure 2B,C, red panel) and colocalized with UTX or JMJD3 (Figure 2B,C, merge panel) at many nuclear foci. This further suggests that ORF59 associates with UTX and JMJD3 in the reactivated cells.

### 3.3. PAN RNA Mediates ORF59′s Interaction with Demethylases, UTX and JMJD3 

As mentioned above, KSHV PAN RNA has been shown to modulate the chromatin landscape of KSHV’s latent genome by associating with histone demethylases, UTX and JMJD3 and activating lytic replication. Once the interaction between ORF59 and UTX or JMJD3 was confirmed, we next evaluated the possible role of PAN RNA during ORF59′s interaction with these H3K27me3 demethylases, UTX and JMJD3. 

**Figure 3:**
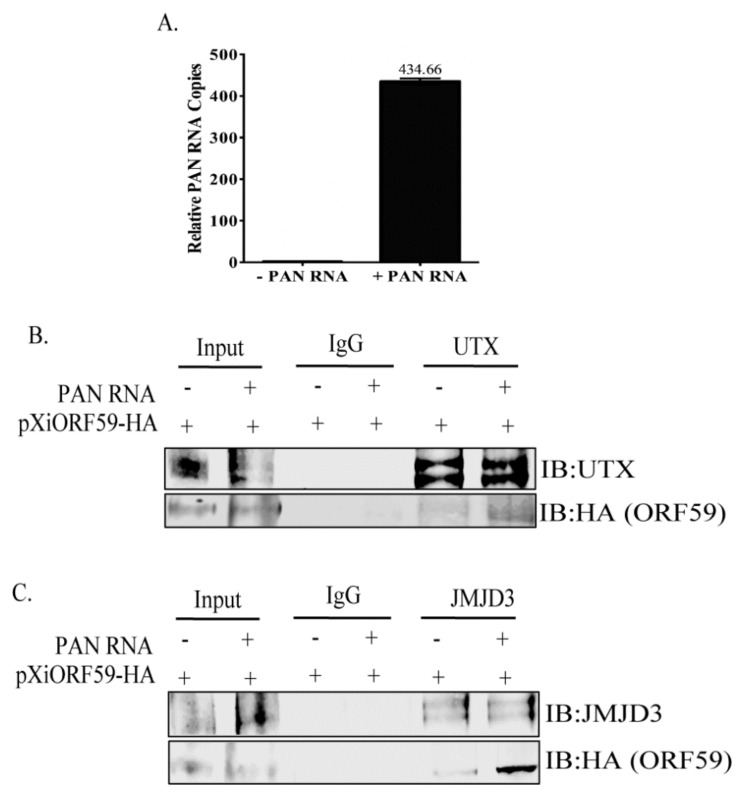
PAN RNA expression enhances ORF59′s interaction with UTX or JMJD3. (**A**) Total RNAs extracted from HEK293 cells transfected with or without PAN RNA and the expression vectors were subjected to RT-qPCR to determine the level of PAN RNA transcripts. Co-IPs were performed on HEK293 cells transfected with or without PAN RNA and the specific expression vectors. (**B**) IP with anti-UTX antibody showed ORF59 co-precipitated in the presence of PAN RNA only (lane 6). (**C**) IP with anti-JMJD3 antibody displayed more ORF59 binding activity in the presence of PAN RNA (lane 6) than in the absence of PAN RNA (lane 5). Control IgG antibody was used as a control.

To investigate the role of PAN RNA in ORF59–demethylase interactions, Co-IP assays were performed on HEK293 cells co-transfected with plasmids expressing ORF59, UTX or JMJD3, in the presence or absence of PAN RNA expressing plasmid using control IgG, UTX or JMJD3-specific antibodies. Efficient expression level of transiently transfected PAN RNA was confirmed by extracting the total RNA from the cells and comparing it with the empty vector transfected cells (Figure 3A). We hypothesized if PAN RNA affected the binding between ORF59 and UTX or JMJD3, the cells with PAN RNA would have enhanced levels of coprecipitating ORF59 from the PAN RNA-expressing cells in comparison to the cells that lack PAN RNA. As expected, IP with anti-UTX and subsequent detection of ORF59 showed an enhanced coprecipitation of ORF59 protein in the presence of PAN RNA (Figure 3B, lane 5 shows limited ORF59 in the absence of PAN RNA, lane 6 shows enhanced ORF59 in the presence of PAN RNA). The lack of an ORF59 band with control IgG antibody indicated the specificity of the ORF59′s interaction with UTX (Figure 3B, lanes 3–4). Additionally, IP with anti-JMJD3 followed by detection of ORF59 showed pronounced ORF59 binding activity in the presence of PAN RNA (Figure 3C, lane 6), compared to in the absence of PAN RNA (Figure 3B, lane 5). The lack of ORF59 band with anti-control IgG antibody indicated the specificity of the ORF59′s interaction with JMJD3 (Figure 3C, lanes 3–4). These results confirm that the interaction between ORF59-UTX and ORF59-JMJD3 is enhanced in the presence of PAN RNA.

### 3.4. ORF59 Enhances the Interaction of PAN RNA with UTX/JMJD3

**Figure 4:**
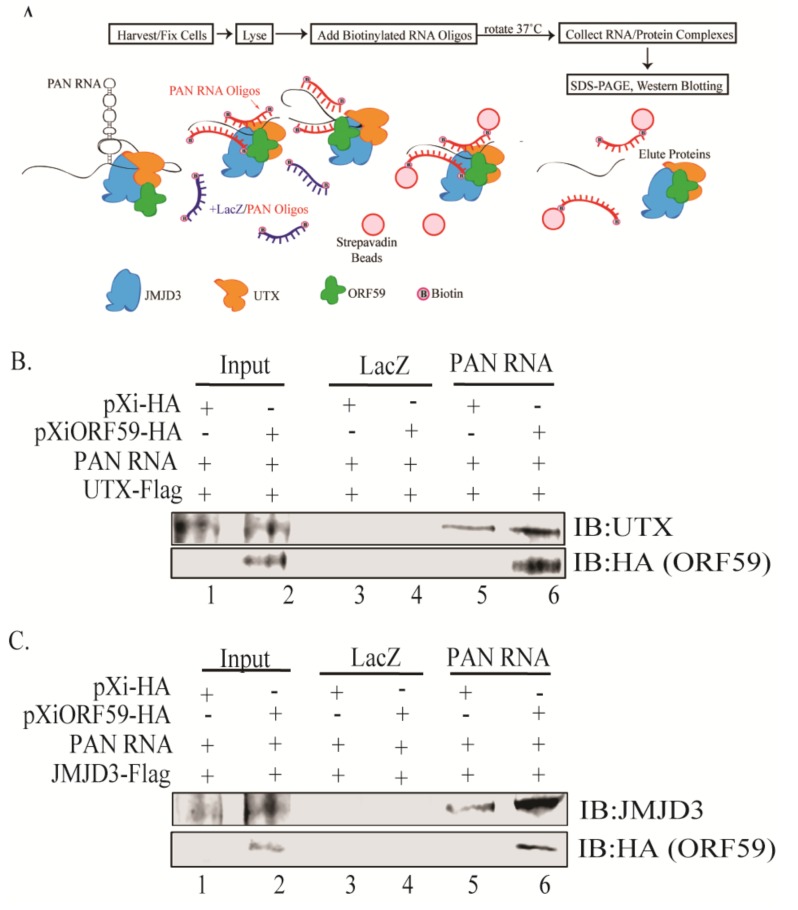
ORF59 protein mediates PAN RNA’s association with UTX or JMJD3. (**A**) Schematic representation of ChIRP assay to isolate PAN RNA-bound proteins. (**B**) HEK293 cells were transfected with PAN RNA and UTX with empty vector or vector expressing ORF59 protein and subjected to ChIRP assay. Expression of ORF59 in cells increased UTX co-precipitation with PAN RNA (lane 6). (**C**) ChIRP assay on HEK293 cells transfected with PAN RNA and JMJD3 with or without ORF59 revealed significantly reduced levels of JMJD3 co-precipitation for ORF59-deficient cells compared to cells expressing ORF59 (lane 6).

**Figure 5:**
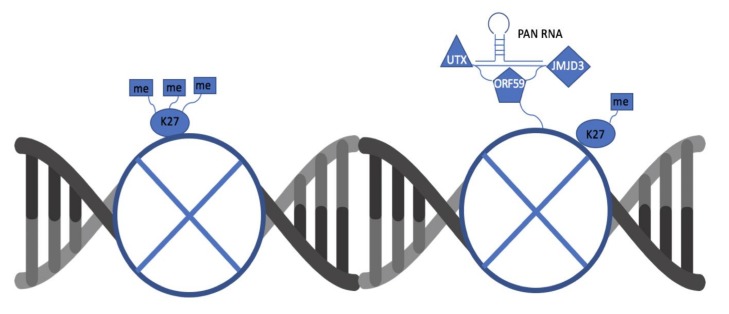
A schematic depicting key interactions between ORF59, PAN RNA, UTX, and JMJD3. The binding of UTX and JMJD3 to ORF59 and PAN RNA is dependent on the presence of both viral factors. This complex is responsible for removing repressive marks on the viral chromatin to promote lytic reactivation.

After confirming the role of PAN RNA in facilitating the ORF59–UTX/JMJD3 protein interactions, we next determined if ORF59 protein also influenced the interaction between PAN RNA and UTX/JMJD3 using chromatin isolation, by an RNA purification (ChIRP) assay that allows isolation of PAN RNA-bound proteins (Figure 4A). To test this, HEK293 cells were transfected with plasmids expressing PAN RNA, UTX or JMJD3 along with either empty or ORF59-expressing plasmids for 24 h. After 24 h, chromatin was cross-linked to preserve PAN RNA-protein adducts and hybridized with either control LacZ or PAN RNA-specific oligos to target PAN RNA, followed by capturing of chromatin complexes using magnetic streptavidin beads and elution of PAN RNA-bound proteins. The eluted protein complexes were run on SDS-PAGE and analyzed by western blotting. If ORF59 influenced the binding between PAN RNA and UTX/JMJD3, then the cells expressing ORF59 should show higher levels of coprecipitated UTX or JMJD3 than ORF59-deficient cells. As expected, UTX or JMJD3 were observed to coprecipitate more in the presence of ORF59, as compared to cells deficient in ORF59 (Figure 4B,C, lane 5–6). Cells lacking ORF59 gene expression served as endogenous positive controls. Lack of ORF59 or UTX/JMJD3 expression for the control LacZ oligos showed that this interaction is specific to PAN RNA (Figure 4B,C, lanes 3–4). Thus, ChIRP analysis showed that presence of ORF59 enhanced the interaction between PAN RNA and UTX/JMJD3. This suggests that ORF59 regulates PAN RNA binding to UTX and JMJD3 and associated chromatin modification of KSHV-repressed genome (Figure 5).

## 4. Discussion

The bi-phasic life cycle of KSHV allows the virus to exist in the infected host without showing any symptoms until triggered to reactivate. Increasing evidence suggests that multiple viral and cellular factors act in concert to reactivate KSHV from latency [29]. Among these, the removal of H3K9me2/me3 and H3K27me3 epigenetic marks commonly found on repressed lytic genes, by chromatin remodeling enzymes, plays a key role in KSHV lytic reactivation [19]. H3K27me3 is reversibly removed through the action of specific histone demethylases, JMJD3 and UTX [30]. Blocking the removal of H3K27me3 by specific inhibition of UTX and JMJD3 by GSK-J4 has been shown to block reactivation in the case of HSV-1 virus [31]. In this study, we explored the mechanism of UTX and JMJD3-mediated chromatin control of KSHV genome.

In the past years, investigation of chromatinized KSHV genome identified PAN RNA’s involvement in altering the state of the viral chromatin to promote lytic reactivation [22]. As a lncRNA, PAN RNA has the ability to regulate gene expression by interacting with chromatin-modifying enzymes, either by direct binding to target specific regions on the genome or by recruiting chromatin modifiers from various sites on the genome [32,33]. lncRNAs can also incorporate into the chromatin-modifying complex to act as a scaffold for assembly. Previous studies have demonstrated that PAN RNA interacts with multiple viral and cellular factors, including UTX and JMJD3, to remove repressive H3K27me3 histone marks allowing increased viral gene expression during KSHV infection. In addition, PAN RNA promoter is directly bound by RTA, a necessary transcription factor for reactivation [34]. MLL2, a methyltransferase also binds to PAN RNA and, in contrast to UTX and JMJD3, MLL2 promotes open chromatin formation by enriching H3K4me3 chromatin marks [25]. PAN RNA can also promote the addition of repressive marks to H3K27me3 by interacting with SUZ12 and EZH2 proteins of the PRC2 complex [27]. By limiting the levels of repressive marks, PAN RNA maintains higher levels of activating marks allowing for the transition to transcriptionally active chromatin and viral gene transcription. Thus, PAN RNA mediates both positive and negative epigenetic regulation. 

Although PAN RNA has been shown to interact with histone modifiers, it remained unclear how PAN RNA could effectively target their activities to specific regions of the genome. Our results provide informative insights into how this mechanism functions, suggesting that PAN RNA is needed for ORF59, the DNA processivity factor’s interaction with UTX and JMJD3. We recently demonstrated ORF59 facilitates chromatin remodeling of the KSHV genome favorable for robust transcription of the lytic genes and production of infectious virions [24]. Co-immunoprecipitations suggest that UTX and JMJD3 are dependent on both ORF59 and PAN RNA being present in order to interact with each viral factor. Previously, PAN RNA knockdown was shown to reduce IE, E, and L gene expression as well as virus production [25,35]. This could partly be explained by the lack of interaction between UTX and JMJD3 with ORF59 in the absence of PAN RNA. It is evident that multifarious PAN RNA acts in a global manner to alter the histone association on chromatin. PAN RNA knockdown studies to provide functional validation and a broader impact of PAN RNA during KSHV reactivation are underway. Identifying these interactions is important to detect newer targets for anti-KSHV treatments and prevent virus from reactivation.
